# Academic stress and performance in first-year health sciences students in Chile: a cross-sectional study

**DOI:** 10.3389/fpsyg.2025.1662109

**Published:** 2025-09-30

**Authors:** Felipe Granada-Granada, Martina Valencia-Narbona, Pablo A. Lizana, Andrea González-Rojas

**Affiliations:** ^1^Laboratorio de Fisiología del Ejercicio, Escuela de Kinesiología, Pontificia Universidad Católica de Valparaíso, Valparaíso, Chile; ^2^Laboratorio de Ciencias Biomédicas Aplicadas a la Kinesiología, Escuela de Kinesiología, Pontificia Universidad Católica de Valparaíso, Valparaíso, Chile; ^3^Laboratory of Epidemiology and Morphological Sciences, Instituto de Biología, Pontificia Universidad Católica de Valparaíso, Valparaíso, Chile

**Keywords:** academic stress, academic performance, first-year students, health sciences education, gender differences, university students, Chile

## Abstract

**Background:**

Academic stress can negatively influence academic performance, particularly in health science programs with high demands.

**Objective:**

To analyze the association between perceived academic stress and academic performance in first-year kinesiology students, considering gender differences and stress dimensions (stressors, reactions, and coping strategies). Methods: This cross-sectional study included 82 students assessed using the SISCO-IIA inventory. Academic performance was measured via final course grades. Descriptive analyses, chi-square tests, *t*-tests, and binary logistic regression models were applied.

**Results:**

Students with high levels of academic stressors were significantly more likely to underperform (OR = 4.62, 95% CI [1.24, 17.22], *p* = 0.023). Female students reported higher emotional and behavioral responses to stress but were less likely to underperform (OR = 0.30, 95% CI [0.11, 0.79], *p* = 0.014). Age was not significantly associated with performance (*p* = 0.433).

**Conclusion:**

Academic stress negatively influences academic outcomes among first-year students. Gender differences in stress response patterns may affect vulnerability and coping effectiveness. These findings highlight the need for early screening, targeted interventions, and support systems to mitigate academic stress and promote student success.

## 1 Introduction

Academic stress (AS), a common experience among university students, has been shown to significantly impact academic performance and is regarded as one of the most frequently reported psychological challenges in higher education worldwide (Brata et al., 2025). Its effects on student performance and overall well-being have received increasing attention in recent years, particularly in the aftermath of the COVID-19 pandemic (González et al., 2023; Kashif et al., 2024; Prifti and Rapti, 2018). This multifaceted nature of AS aligns with the bio-psycho-social paradigm (World Health Organization, n.d.), which highlights the integration of biological, psychological, and social components in understanding stress. AS can be defined as the psychological response to academic demands that exceed an individual’s perceived ability to cope effectively, often resulting in emotional distress, cognitive fatigue, and reduced academic outcomes (Castillo-Navarrete et al., 2020). Stress associated with academic demands has been linked to lower academic performance, diminished motivation, and a heightened likelihood of experiencing mental health issues, including anxiety, depression, sleep disturbances, and substance use (Córdova Olivera et al., 2023; Pascoe et al., 2020). Research has identified stress levels as a key factor influencing academic outcomes, with high levels of perceived stress correlating with poorer mental health and academic failure (Amhare et al., 2021; Javaid et al., 2024). For example, (Almarzouki, 2024) found that stress can impair cognitive processes such as working memory, which plays a fundamental role in academic performance, thereby potentially undermining students’ academic capabilities. In summary, high-stress levels among university students are associated with persistent mental health issues and can affect their academic performance (Stirparo et al., 2024).

In Chile, the mental health of university students has emerged as a pressing public health concern, as this population is particularly vulnerable to developing mental health disorders (Martínez-Líbano et al., 2023). The transition from secondary to higher education is a critical period characterized by intense academic and personal adjustments (Cifuentes Gomez et al., 2022). During this stage, students must navigate new academic demands, manage their time effectively, and adopt unfamiliar study strategies, challenges that are often compounded by structural inequalities (Naylor et al., 2021). Factors such as social class, migration status, disability, and the mismatch between expectations and academic reality further exacerbate disparities in adaptation (Almeida et al., 2022; Easterbrook and Hadden, 2021; Naylor et al., 2021). In this context, support from family, peers, and educational institutions becomes essential to promote a successful transition (Estefan et al., 2023).

A common challenge is the mismatch between students’ expectations and the actual experience of higher education, which often results in stress and difficulties adapting (Cifuentes Gomez et al., 2022; Duarte and Sandoval, 2017). Additionally, this transition is marked by feelings of instability and uncertainty, especially among first-generation and low-income students (Duarte and Sandoval, 2017; Garcia and Wood, 2023; Palma-Amestoy, 2022). Social class also shapes students’ aspirations and experiences—while those from privileged backgrounds tend to view university as a natural path, others face uncertainty and social pressure (Duarte and Sandoval, 2017; Garcia and Wood, 2023; Palma-Amestoy, 2022). Family and peers serve as primary sources of information and support, significantly influencing students’ expectations and coping strategies during this period (Cifuentes Gomez et al., 2022; Garcia and Wood, 2023; Guzmán et al., 2021).

Despite policies such as tuition-free education, access to university for first-generation and technical program students has seen only modest gains. This suggests that financial support alone is insufficient to address deeper structural inequalities in access to higher education (Aldinucci et al., 2023; Espinoza et al., 2022). Migrant students face additional barriers; however, those who enter the Chilean school system before the age of 10 tend to follow higher education trajectories similar to native students (Abufhele et al., 2024). Adult support and institutional mechanisms act as key protective factors for this population (Abufhele et al., 2024; Díaz Pacheco et al., 2023).

On the other hand, first-year university students are frequently exposed to unfamiliar teaching methods, heightened academic expectations, and less structured environments. These challenges often occur alongside increased personal autonomy and the demands of relocation. Such pressures have been shown to contribute to high levels of stress and increased risk of academic failure or dropout, particularly during the first two semesters (Cifuentes Gomez et al., 2022; Guzmán et al., 2021).

This situation is especially relevant for students in health-related academic programs, such as medicine, nursing, and kinesiology, fields with extensive content, and can significantly impact their overall development during their education (Pérez Briones et al., 2023). Heavy course loads, early exposure to clinical content, and high-performance expectations characterize these programs. Studies conducted with Chilean students in health sciences have reported elevated rates of academic stress, with contributing factors including performance anxiety, fear of failure, limited time for personal activities, and the competitive nature of these programs (Castillo et al., 2018; Martínez-Líbano et al., 2023; Reverté-Villarroya et al., 2021). Notably, stress levels tend to be higher in female students and those with fewer academic coping resources (Graves et al., 2021; Imran et al., 2025) Several studies have shown that women tend to report significantly higher levels of academic stress compared to men, particularly in the dimensions of physical and psychological reactions, as well as in the perception of academic stressors (Graves et al., 2021; Imran et al., 2025). These differences are also reflected in coping strategies, with women more frequently using emotion-focused and social support strategies, while men tend to rely on more instrumental approaches (Castillo-Navarrete et al., 2023; Fonseca-Molina et al., 2018).

Academic performance, often measured through grades or course completion, is a complex outcome affected by psychological, cognitive, and social variables (Hachem et al., 2022; Shi and Qu, 2021; Tindle et al., 2022). Studies show that AS may negatively impact academic performance, though findings are often moderated by gender, age, and coping strategies (Frazier et al., 2019; Pascoe et al., 2020; Rani, 2025; Zhang et al., 2024). While some evidence suggests that younger students may face more adjustment difficulties, age-related patterns remain understudied (Belay Ababu et al., 2018; Espinoza et al., 2025; Mudhovozi, 2012; Páramo Fernández et al., 2017).

Despite growing awareness, research that examines the relationship between academic stress and academic performance in Chilean university students—especially using psychometrically validated tools—is still scarce. The SISCO Inventory for Academic Situations (SISCO-IIA) offers a comprehensive measure of academic stress, evaluating stressors, physiological and emotional reactions, and coping strategies (Castillo et al., 2018; Castillo-Navarrete et al., 2024; Guzmán-Castillo et al., 2022). Importantly, the instrument has been validated for Chilean university populations and includes gender-specific normative data, making it a culturally and contextually appropriate tool for educational research in this setting (Castillo et al., 2018; Castillo-Navarrete et al., 2024; Guzmán-Castillo et al., 2022).

Although the relationship between academic stress and academic performance has been widely studied, most research has focused on general university populations or disciplines outside the health sciences, often within North American, European, or Asian contexts (Frazier et al., 2019; Iqbal et al., 2021; Kötter et al., 2017; Pascoe et al., 2020). Few studies have examined this association in Latin America, particularly in Chile, where first-year health sciences students face distinct academic and cultural challenges (Chacon et al., 2025; Espinoza et al., 2025). These students often transition directly from secondary education into highly demanding programs that include early exposure to clinical environments and competitive academic pressures, all within a context of socioeconomic inequalities and strong familial expectations (Delgado-Floody et al., 2020; Espinoza et al., 2025). These contextual factors may influence both stress responses and academic outcomes in ways that differ from those observed in other regions. Therefore, the objective of this study is to examine the association between academic stress and academic performance in first-year kinesiology students at a Chilean university, considering stress dimensions and controlling for age and gender. We hypothesize that higher levels of academic stress are associated with lower academic performance, and that gender differences modulate both stress responses and academic outcomes.

By focusing on a high-risk academic transition period and employing a culturally validated stress inventory with analytical refinements, this research contributes to a deeper understanding of how academic stress affects student achievement and retention in the health sciences. Additionally, our study uses a multidimensional measure of academic stress (SISCO-IIA), incorporates gender-specific normative thresholds for stress classification, and controls for age and gender—methodological elements not commonly addressed in prior research. Building on the findings of this study, institutions can develop more effective strategies for the early identification of students at risk, implement tailored academic support measures, and promote overall well-being, particularly among vulnerable student populations facing high academic demands.

## 2 Materials and methods

### 2.1 Research design and participants

This cross-sectional, observational study examined the association between AS and academic performance among first-year university students. The sample was drawn through non-probabilistic, convenience sampling. It included students enrolled in the course “Health Promotion and Self-Care” during their second academic semester in the Kinesiology program at the Pontificia Universidad Católica de Valparaíso in 2023. Inclusion criteria were enrollment in the course and being a first-time kinesiology student. Students were excluded if they did not complete the semester or had previously studied in another university program.

A total of 110 students were initially enrolled. After excluding seven students from the baccalaureate program, eight repeating students, and 13 who did not respond to the survey, the final sample consisted of 82 students. Demographic data collected included age, and gender (male, female, other).

### 2.2 Measures

#### 2.2.1 Academic stress (AS)

Academic stress was measured using the SISCO Inventory for Academic Situations (SISCO-IIA), a validated instrument specifically designed to assess academic stress in higher education students (Castillo-Navarrete et al., 2020; Guzmán-Castillo et al., 2022). The inventory consists of 32 items: one dichotomous item assessing the presence or absence of academic stress, and 31 items rated on a 5-point Likert scale ranging from 1 (“not at all” or “very low”) to 5 (“very much” or “very high”). These items evaluate the frequency and intensity of academic stressors, cognitive-emotional responses, and coping strategies in academic contexts. SISCO-IIA provides a total stress score by summing the Likert-scale items, with higher scores indicating greater levels of perceived AS.

Based on established gender-specific normative data for Chilean university students, total scores were categorized into low, medium, and high AS. For female students, scores ≤86 indicated low AS, 87–107 indicated medium AS, and ≥108 indicated high AS; for male students, scores ≤78 indicated low AS, 79–98 indicated medium AS, and ≥99 indicated high AS (Castillo-Navarrete et al., 2024). The instrument has demonstrated strong internal consistency and construct validity within Chilean samples. In the current study, the internal consistency of the Academic Stress Inventory was assessed using Cronbach’s alpha. The total scale demonstrated high internal consistency, with a Cronbach’s alpha coefficient of 0.77344.

#### 2.2.2 Procedure

Data collection was conducted on September 23, 2024, during the second academic semester. Prior to the beginning of a scheduled class session, students were informed about the objectives of the study and invited to participate voluntarily. Those who agreed signed an informed consent form, in accordance with the ethical standards of the Pontificia Universidad Católica de Valparaíso and the Declaration of Helsinki.

Participants received a printed survey package, which included a sociodemographic questionnaire and the SISCO-IIA (Castillo-Navarrete et al., 2020; Guzmán-Castillo et al., 2022). The instruments were administered in person, in a supervised classroom setting, ensuring a quiet environment that minimized distractions. Students were given a maximum of 15 min to complete the materials individually. The researchers remained available throughout the session to clarify any doubts or procedural questions.

All questionnaires were collected immediately after completion to ensure data integrity and confidentiality. No incentives were provided, and participation or non-participation had no impact on students’ academic evaluation or course progress.

#### 2.2.3 Academic performance

Academic performance was measured using the final grade of the course, based on a 1.0 to 7.0 grading scale (passing threshold: 65%). For analysis purposes, performance was dichotomized using the 50th percentile (p50) as the cutoff. This measure was obtained at the end of the academic semester, once the course had been completed.

### 2.3 Ethical considerations

All procedures followed the ethical standards of the Declaration of Helsinki for research involving human participants (World Medical Association, 2025) and the institutional guidelines approved by the Bioethics Committee of the Pontificia Universidad Católica de Valparaíso (approval code: BIOEPUCV-H 693-2023). Informed consent was obtained from all participants.

### 2.4 Statistical analysis

All statistical analyses were performed using Stata Statistical Software: Release 16 (StataCorp, 2019). Descriptive statistics included frequencies and percentages for categorical variables and means with standard deviations (M ± SD) for continuous variables. The Kolmogorov–Smirnov test assessed normality. Chi-square and Fisher’s exact tests were used for associations between categorical variables, and *t*-tests or Mann–Whitney U tests were used for mean comparisons, depending on data distribution. Specifically, the Mann–Whitney U test was applied to compare age distributions between male and female students, showing no significant difference (*p* = 0.996). The chi-square test was also used to examine differences in academic stress levels (e.g., stressors, reactions) between sexes.

For inferential analysis, low and medium AS levels were grouped and compared with the high AS group. Logistic regression models specifically binary logistic regression was used to assess the association between high academic stress and low academic performance (defined as a final grade below the 50th percentile). Given the dichotomous nature of the outcome variable, binary logistic regression was appropriate. Crude models examined the association of each AS dimension independently, and adjusted models included gender and age as covariates. Odds ratios (ORs), 95% confidence intervals (CIs), and *p*-values were reported. Model fit was evaluated using the Hosmer-Lemeshow test. Statistical significance was set at *α* = 0.05.

## 3 Results

### 3.1 General characteristics of the sample

Table 1 presents the descriptive characteristics of the participants. Responses from 82 students were analyzed, of whom 45 were male and 37 were female. The average age for male students was 18.70 ± 0.74 years, and for female students, 18.71 ± 0.75 years, with no significant differences between the groups (*p* = 0.99; Mann–Whitney test).

**Table 1:** General characteristics of the participants.

Variable	Category/statistic	*n* (%)/M (±SD)
Age (years)	Mean (±SD)	18,71 ± 0,75
Gender	Female	37 (45.1%)
	Male	45 (54.9%)
Academic stress (present)	Yes	80 (97.6%)
	No	2 (2.4%)
Academic stress level general (among those who reported stress)	Low-medium	25 (31.6%)
	High	54 (68.3%)
Academic stress level female	Low-medium	10 (12.6%)
	High	25 (31.6%)
Academic stress level male	Low-medium	15 (18.9%)
	High	29 (30.7%)
Academic performance general (final grade)	High (≥ p50)	69 (84.1%)
	Low (< p50)	13 (15.8%)
Academic performance female (final grade)	High (≥ p50)	33 (94.3%)
	Low (< p50)	2 (5.7%)
Academic performance male (final grade)	High (≥ p50)	37 (78.7%)
	Low (< p50)	10 (21.2%)

Variables are presented as means with standard deviations for continuous data, and as the number of students per condition with the corresponding percentage relative to the total of each group for categorical data.

Among the participants, 97.6% (*n* = 80) reported experiencing academic stress. Of those who reported stress, 67.9% (*n* = 53) exhibited a high level of stress, while 32.1% (*n* = 25) showed low or medium levels.

The average academic performance score was 5.18 (SD = 5.00) on a scale from 1.0 to 7.0. Academic performance was categorized based on the 50th percentile (P50) of the final course grade. According to this classification, 84.1% of the students (*n* = 69) achieved a high academic performance (final grade ≥ P50), while 15.8% (*n* = 13) obtained a low academic performance (final grade < P50) (Table 1).

### 3.2 Gender differences in academic stress dimensions

When exploring gender differences, female students obtained slightly higher final course grades (5.29 ± 3.57) than male students (5.09 ± 5.7), although this difference was not statistically significant (Student’s *t*-test, *p* = 0.0582). Table 2 summarizes the distribution of academic stress levels across the five dimensions of the SISCO-II-AS scale by gender. Statistically significant differences were found between male and female students in four out of six comparisons. Specifically, females exhibited higher stress levels than males in the dimensions of physical and psychological reactions (χ^2^ = 10.61, *p* = 0.005), social behavioral reactions (χ^2^ = 12.11, *p* = 0.002), total reaction (χ^2^ = 12.31, *p* = 0.002), and the full instrument (χ^2^ = 10.02, *p* = 0.007). These findings are supported by the observation that a greater proportion of female students reported high stress levels in these dimensions compared to males. In contrast, no significant gender differences were observed in the stressors (χ^2^ = 1.37, *p* = 0.505) or coping strategies (χ^2^ = 0.76, *p* = 0.683) dimensions. These results suggest that although both genders experience academic stress, female students tend to report more intense emotional and behavioral stress responses.

**Table 2:** Summary of the standards set for SISCO-II-AS (full instrument and its subscales).

Academic stress subscales	Male (*n* = 45)	Female (*n* = 37)	χ^2^	*p*-value
Stressors				
Low	19 (42.22)	11 (29.73)	1.3658	0.505
Medium	20 (44.44)	20 (54.5)		
High	6 (13.33)	6 (16.22)		
Physical and psychological reactions				
Low	22 (48.89)	6 (16.22)	10.6069	**0.005**
Medium	15 (33.33)	24 (64.86)		
High	8 (17.78)	7 (18.92)		
Social behavioral reactions				
Low	22 (48.89)	5 (13.51)	12.1051	**0.002**
Medium	18 (40.00)	22 (59.46)		
High	5 (11.11)	10 (27.03)		
Total reaction				
Low	27 (60.00)	8 (21.62)	12.3137	**0.002**
Medium	11 (24.44)	19 (51.35)		
High	7 (15.56)	10 (27.03)		
Coping strategies				
Low	11 (24.44)	11 (29.73)	0.7621	0.683
Medium	22 (48.89)	19 (51.35)		
High	12 (26.67)	7 (18.92)		
Full instrument				
Low	25 (55.56)	8 (21.62)	10.0215	**0.007**
Medium	13 (28.89)	21 (56.76)		
High	7 (15.56)	8 (21.62)		

Variables are presented as the number of students per condition and the percentage relative to the total of each group. Analyses by variable were conducted using the Chi-square test (χ^2^). *p* < 0.05. Chi-square tests (χ^2^) were used to assess global differences in the distribution of academic stress levels (low, medium, high) between sexes for each dimension. These values do not represent comparisons at each individual stress level. Post hoc analyses can be provided upon request. *p*-values < 0.05 are shown in bold.

### 3.3 Association between academic stress and academic performance

When examining the association between academic stress and performance, students with lower academic performance (<p50) exhibited significantly higher levels of academic stress compared to their higher-performing peers (≥p50) (*p* = 0.047). Further analysis by stress dimensions revealed that low-performing students reported higher frequencies of elevated physical and psychological reactions (27.5% vs. 9.5%; *p* = 0.047), total reaction (30.0% vs. 11.9%; *p* = 0.043), and in the full instrument score (27.5% vs. 9.5%; *p* = 0.047). These findings suggest that greater exposure to and difficulty coping with academic stress is associated with poorer academic outcomes, particularly in the domains of emotional and physiological response (Tables 3, 4).

**Table 3:** Association between academic performance with high academic stress and sociodemographic characteristics of university students.

Academic stress and sociodemographic characteristics	Final averages (≥p50) *n*, (%)	Final averages (<p50) *n*, (%)	*p*-value
Gender			
Male	18 (42.86)	27 (67.50)	**0.025**
Female	24 (57.14)	13 (32.50)	
Age (years) mean ± SD	18.8 ± 0.6	18.7 ± 0.8	0.221
High-stressors	3 (7.1)	9 (22.5)	0.064
Low/Moderate-stressors	39 (92.9)	31 (77.5)	
High-physical and psychological reactions	4 (9.5)	11 (27.5)	**0.047**
Low/moderate-physical and psychological reactions	38 (90.5)	29 (72.5)	
High-Social behavioral reactions	6 (14.3)	9 (22.5)	0.336
Low/moderate-social behavioral reactions	36 (85.7)	31 (77.5)	
High-total reaction	5 (11.9)	12 (30.0)	**0.043**
Low/moderate-total reaction	37 (88.1)	28 (70.0)	
High-coping strategies	10 (23.8)	9 (22.5)	0.888
Low/moderate-coping strategies	32 (76.2)	31 (77.5)	
High-full instrument	4 (9.5)	11 (27.5)	**0.047**
Low/moderate-full instrument	38 (90.5)	29 (72.5)	

Variables are presented as the number of students per condition and the percentage relative to the total in each group. Analyses by variable were conducted using the Chi-square test (χ^2^) and Fisher’s exact test, as appropriate. Statistical significance was set at *p* < 0.05. *p*-values < 0.05 are shown in bold.

**Table 4:** Logistic regression for the association between final averages and academic stress in university students.

Academic stress and subscale, gender and age	Final averages (<p50)		Final averages (<p50)	
	OR [95%CI]	*p*	OR [95%CI]	*p*
High-stressors	3.77 [0.94–15.14]	**0.061**	4,28 [0.98–18.5]	0.053
Gender (female)			0.32 [0.13–0.83]	**0.019**
Age (years)			0.91 [0.49–1.71]	0.775
High-Physical and psychological reactions	3.60 [1.04–12.48]	**0.043**	4.09 [1.12–14.92]	**0.033**
Gender (female)			0.33 [0.13–0.84]	**0.021**
Age (years)			0.78 [0.41–1.48]	0.441
High-social behavioral reactions	1.74 [0.56–5.44]	0.340	2.71 [0.78–9.40]	0.116
Gender (female)			0.29 [0.11–0.77]	**0.013**
Age (years)			0.75 [0.39–1.42]	0.372
High-Total reaction	3.17 [1.00–10.05]	0.050	4.63 [1.31–16.29]	**0.017**
Gender (female)			0.27 [0.10–0.73]	**0.010**
Age (years)			0.75 [0.39–1.45]	0.391
High-coping strategies	0.93 [0.33–2.59]	0.888	**0.87 [1.24–17.22]**	0.793
Gender (female)			0.35 [0.14–0.88]	**0.025**
Age (years)			0.81 [0.44–1.50]	0.500
High-full instrument	3.60 [1.04–12.48]	**0.043**	4.62 [1.24–17.22]	**0.023**
Gender (female)			0.30 [0.11–0.79]	**0.014**
Age (years)			0.77 [0.40–1.48]	0.433

The models were evaluated using the Hosmer-Lemeshow test (*p* > 0.05). *p*-values < 0.05 are shown in bold.

### 3.4 Binary logistic regression models

Binary logistic regression models were conducted to examine the association between high levels of academic stress (in various dimensions) and the likelihood of presenting lower academic performance (final grade <p50) (Table 4). The models showed that high levels of physical and psychological reactions significantly increased the odds of lower academic performance (OR = 4.09, 95% CI: 1.12–14.92, *p* = 0.033), as did high total stress reaction (OR = 4.63, 95% CI: 1.31–16.29, *p* = 0.017) and high stress as measured by the full SISCO-II-AS instrument (OR = 4.62, 95% CI: 1.24–17.22, *p* = 0.023). Being female was consistently associated with lower odds of poor performance across most models (e.g., OR = 0.30, 95% CI: 0.11–0.79, *p* = 0.014). Age was not a significant predictor in any model. These results suggest that specific components of academic stress—particularly the physical, emotional, and overall responses—are significantly associated with decreased academic performance in university students.

## 4 Discussion

This study explored the relationship between perceived academic stress and academic performance among first-year kinesiology students in Chile. Understanding this association is particularly important in the context of university transition, a critical period marked by increased academic demands, emotional adjustment, and the development of autonomous learning habits. Identifying how stress impacts academic outcomes in early stages of higher education can inform targeted support strategies that enhance student retention and success, especially in demanding health-related programs. We hypothesized that higher levels of academic stress would be associated with lower academic performance, and that gender differences would modulate both stress responses and academic outcomes. The findings support this hypothesis: students reporting high levels of academic stressors were significantly more likely to exhibit lower final course grades, even after adjusting for gender and age. Although male students represented a larger proportion of the sample, a significantly higher percentage of female students reported medium and high levels of stress in the subscales of physical and psychological reactions, social behavioral responses, and overall stress reaction. These findings suggest that while both genders may experience similar academic demands, women tend to exhibit more intense emotional and physiological responses to those stressors. This pattern aligns with existing literature that documents greater emotional reactivity and the use of emotion-focused coping strategies among female students. Therefore, the observed gender differences in stress responses may partially explain the protective effect of being female on academic performance found in this study. These results underscore the importance of considering not only the intensity but also the qualitative aspects of academic stress—such as coping mechanisms and emotional responses—when designing targeted interventions to support students during the transition into higher education.

These findings reinforce previous evidence showing that academic stress, particularly when left unaddressed, is a measurable risk factor for academic underperformance and increased psychological vulnerability during the university transition period (Castillo-Navarrete et al., 2024; Martínez-Líbano et al., 2023). Crucially, the data underscore the multidimensional nature of academic stress. The SISCO-IIA inventory allowed us to analyze distinct dimensions—stressors, reactions, and coping strategies—demonstrating that stress is not a uniform experience. Students interpret and react to academic demands differently, and the effectiveness of their coping strategies appears to play a decisive role in shaping their academic trajectories. This complexity mirrors the biopsychosocial model of stress, which considers interactions between individual perceptions, physiological responses, and contextual demands (Castillo-Navarrete et al., 2024; Guzmán-Castillo et al., 2022).

Moreover, the analysis of coping strategies revealed meaningful differences between students who reported high versus low academic stress levels. Emotion-focused coping—such as rumination, avoidance, or anxiety—was more frequently used by students experiencing high stress, whereas those with lower stress scores reported greater use of adaptive strategies, such as planning, time management, and seeking academic or emotional support. These findings align with existing evidence indicating that maladaptive coping strategies tend to exacerbate the impact of academic stress, particularly when students feel unprepared or lack institutional support (Cabanach et al., 2015; Fonseca-Molina et al., 2018). Therefore, strengthening adaptive coping mechanisms during the first year may act as a buffer against the negative impact of academic stress on performance and psychological well-being.

Recent neurobiological studies offer a compelling framework to interpret these findings. Academic stress activates the hypothalamic–pituitary–adrenal (HPA) axis, increasing cortisol secretion—a hormone known to impair memory, attention, and executive functioning (Anjum et al., 2021; Jamieson et al., 2021). These cognitive domains are essential for learning and academic performance, particularly in rigorous programs such as health sciences, where content is dense and applied learning is required from early stages. Furthermore, elevated stress has been associated with reductions in alpha wave activity and increases in delta wave activity in the left temporal lobe—an area central to memory and learning (Cronje et al., 2024). At the molecular level, stress-induced reductions in brain-derived neurotrophic factor (BDNF) and increases in global deoxyribonucleic acid (DNA) methylation (5-mC) have been linked to diminished neuroplasticity and synaptic function (Castillo-Navarrete et al., 2024), offering a biological explanation for cognitive disruption under stress. These mechanisms may explain why a notable portion of our sample, almost 20%, reported high stress and underperformed academically. Moreover, biomarkers such as elevated salivary protein and cortisol levels are increasingly recognized as reliable indicators of AS in university settings (Brata et al., 2025; Zallocco et al., 2021). Complementary findings suggest that vitamin D deficiency, which impairs neurotransmission and neuroplasticity, is also associated with stress and anxiety symptoms that may influence academic outcomes (Ibrahim and Audi, 2021).

Interestingly, our study revealed the protective effect of gender. Despite previous literature identifying female students as more prone to report stress and anxiety (Martínez-Líbano et al., 2023), women in our sample were less likely to fall below the academic performance median. This finding may reflect the greater use of adaptive coping strategies among women, such as emotional regulation and help-seeking behaviors (Cabanach et al., 2015; Fonseca-Molina et al., 2018), which can buffer the impact of stress on academic performance. Conversely, male students—who were overrepresented in the low-performing group—may underreport emotional distress or avoid support systems, amplifying the negative effects of stress (Castillo et al., 2018). Age, in contrast, did not significantly predict academic outcomes—likely due to the relative homogeneity in age within the sample. However, age may become more relevant in diverse or longitudinal cohorts.

The findings observed must be interpreted considering the multiple demands that students face during their transition into higher education, particularly in programs such as kinesiology and health sciences. This period is marked by increased academic rigor, emotional adjustment, and the development of autonomous learning strategies. These stressors can overwhelm students’ coping capacities, especially if they lack previous exposure to high academic demands. Our results reflect this dynamic: high perceived stress levels, especially in the psychological and physiological domains, were significantly associated with poorer academic performance. This highlights the need for universities to implement support systems that consider the psychosocial adaptation process during students’ first year.

Although age is commonly considered a factor influencing academic adaptation, especially during transitions to higher education, our study did not find a significant association between age and academic performance. This result is likely attributable to the homogeneity of the sample in terms of age distribution. Specifically, most students in our sample were recent high school graduates entering university directly, which results in a narrow age range and minimal developmental variability. Under such conditions, age-related differences in cognitive maturity, emotional regulation, or prior academic experience may not be sufficiently distinct to produce measurable differences in academic outcomes. Moreover, previous research has shown that the predictive value of age on academic performance is more prominent in heterogeneous samples that include non-traditional students, such as those returning to education later in life or combining study with work or family responsibilities, whose life experiences and learning strategies differ substantially from those of traditional students (Kasworm and Pike, 1994; Spitzer, 2000; Wambugu and Emeke, 2019). In contrast, within cohorts of first-year students in health-related programs, such as kinesiology, age tends to be a poor differentiator of academic trajectories unless accompanied by other moderate variables (e.g., socioeconomic background, previous academic preparation, or learning disabilities) (Fernández-Galván et al., 2024; Slater and Cusick, 2017). Therefore, in the context of this study, age likely does not function as a strong predictor due to the relatively uniform academic background and developmental stage of the participants. Future studies could benefit from including more age-diverse samples or examining the interaction between age and other psychosocial variables to better understand its role in academic performance.

A major strength of this study lies in its use of a culturally validated instrument tailored to the Chilean university context. SISCO-IIA provided nuanced insights into students’ stress profiles, offering a more accurate and context-sensitive assessment than generic instruments. Nonetheless, limitations should be noted. Additionally, the study presents some limitations that may affect the scope and interpretation of its findings. The relatively small sample size restricts the statistical power of subgroup comparisons and limits the generalizability of results to broader student populations or disciplines. The cross-sectional design also prevents establishing causal relationships between stress dimensions and academic outcomes. Furthermore, another limitation of the present study is the absence of a formal *post hoc* statistical power analysis for the comparisons performed. We acknowledge that this omission may restrict the interpretation of the true magnitude of the observed findings, particularly in analyses involving small subsample sizes. Future research, especially studies with analytical or inferential aims, should complement their results with more precise effect size estimates and incorporate statistical power analyses to strengthen the interpretation of outcomes. Moreover, we recommend that future studies include, from the design stage, a prospective calculation of sample size and statistical power to ensure greater methodological robustness.

### 4.1 Practical implications and recommendations

The present findings offer several practical implications for higher education institutions, particularly those delivering health science programs where academic demands are intense from the outset. First, the early identification of students at risk of experiencing high levels of academic stress should be prioritized through the systematic application of validated instruments, such as the SISCO-IIA. Early detection enables the implementation of timely, tailored interventions that may include psychological support services, structured stress management workshops, and academic mentoring programs designed to foster both academic and emotional resilience.

Moreover, institutions should actively promote the development of adaptive coping strategies, especially among male students, who may be less likely to verbalize emotional distress or seek help proactively. Gender-sensitive interventions, such as peer support networks and targeted psychoeducation, could help address this gap and enhance help-seeking behaviors.

Curricular design also plays a key role in mitigating academic stress. Therefore, revisiting program structures to ensure a better balance between academic rigor and student well-being is essential. This may involve the incorporation of flexible and student-centered pedagogical strategies, as well as inclusive assessment methods that reduce perceived pressure and foster a sense of academic self-efficacy.

In parallel, institutions should strengthen existing support systems—including counseling services, peer mentoring initiatives, and faculty training in mental health literacy—to build a more supportive academic ecosystem. Faculty awareness of stress-related challenges and their impact on performance can improve the early referral of at-risk students and facilitate the normalization of mental health discussions within the academic setting.

Finally, future research should expand on these findings by incorporating longitudinal designs, larger and more diverse samples, and the use of objective physiological or neurobiological markers. This would allow for a deeper understanding of the mechanisms linking academic stress to performance and inform the development of evidence-based institutional policies that address academic stress comprehensively and sustainably.

## 5 Conclusion

In conclusion, this study provides evidence of a significant relationship between perceived academic stress and academic performance among first-year health sciences students. The findings highlight the complex and multidimensional nature of academic stress, particularly in transitional academic contexts. The use of a culturally adapted instrument (SISCO-IIA) allowed us to capture not only the presence of stress but also the types of reactions and coping strategies employed by students—dimensions often overlooked in traditional assessments.

Our results suggest that students with higher levels of perceived academic stress, particularly those exhibiting maladaptive emotional or behavioral responses, tend to show lower academic performance. Interestingly, although female students reported higher stress levels across several dimensions, they performed better academically, potentially due to more frequent use of adaptive coping strategies. In contrast, male students were overrepresented in the lower performance group, raising questions about the role of unexpressed stress and underutilization of support systems.

The absence of a significant effect of age may reflect the relative age homogeneity of our sample. However, future research involving more age-diverse or longitudinal samples is needed to explore how academic stress trajectories evolve over time and across different student profiles. Furthermore, the lack of formal *post hoc* power analyses and a modest sample size limit the generalizability of our findings and suggest caution in interpreting small-to-moderate effects.

Despite these limitations, our findings have important practical implications. Institutions should prioritize early screening and targeted interventions to support students, especially in the first year of health-related programs. Promoting adaptive coping mechanisms, integrating mental health resources into the curriculum, and fostering inclusive academic environments may enhance students’ academic success and well-being. Future research should examine longitudinal outcomes, explore biological markers of stress, and evaluate the effectiveness of institutional interventions in reducing academic stress and improving performance.

## Data Availability

The raw data supporting the conclusions of this article will be made available by the authors, without undue reservation.
